# Supplementary material for: The spike gene is a major determinant for the SARS-CoV-2 Omicron-BA.1 phenotype
Source: Nat Commun. 2022 Oct 7;13:5929. doi: 10.1038/s41467-022-33632-y (PMC9543931; doi:10.1038/s41467-022-33632-y)
Supplement: Supplementary file 1 — Supplementary Information [file 41467_2022_33632_MOESM1_ESM.pdf]

Supplementary Figure 1

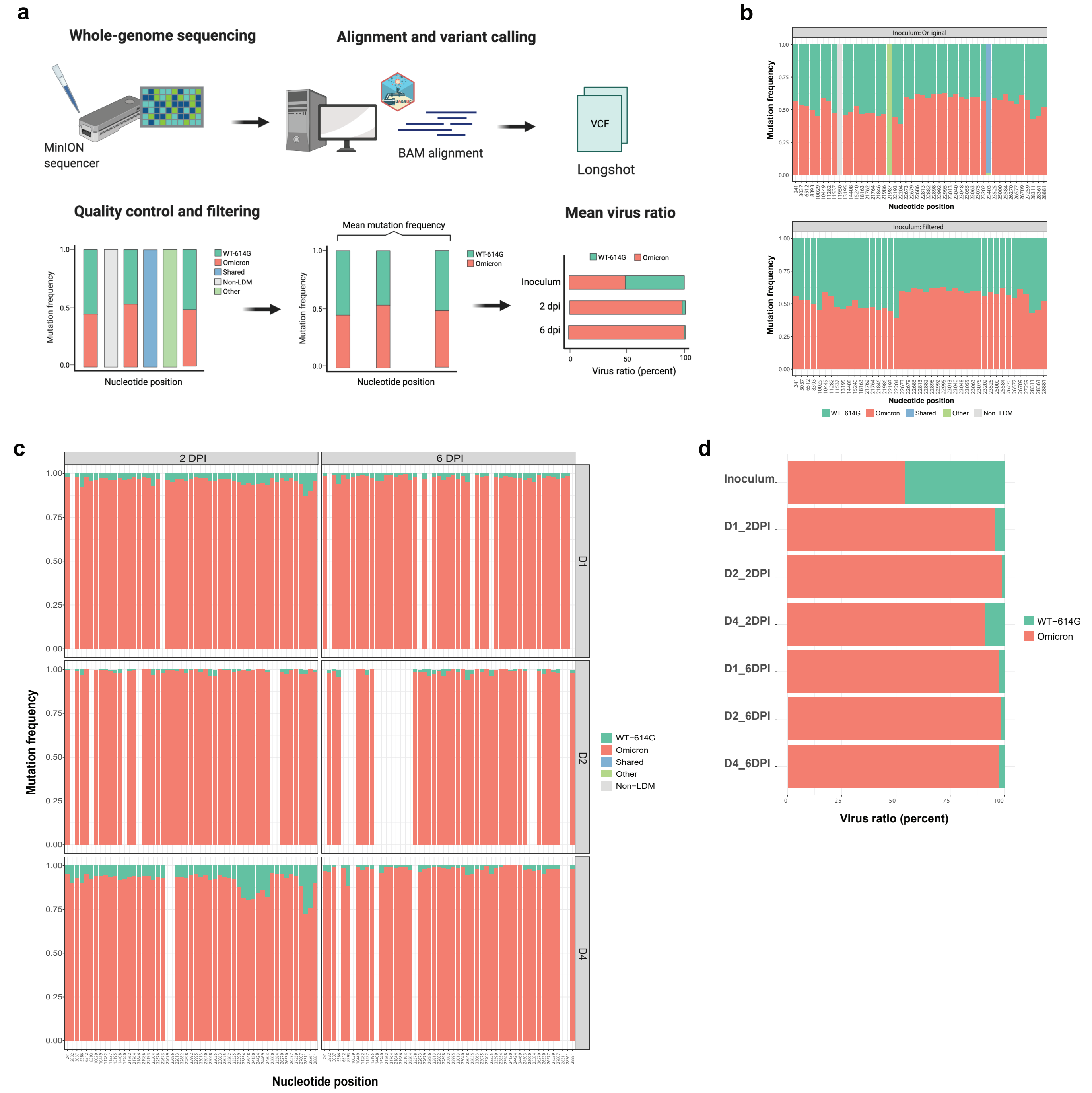

Supplementary Fig. 1: Sequencing data analysis. a) Schematic illustrating the general sequencing and bioinformatics workflow used to determine the virus ratios in Fig. 1e-g. For each virus mixture in the NEC, BEC, and lung explants samples, RNA was extracted from apical washes at 2 and/or 6 dpi and sequenced on the MinION sequencer using a modified version of the ARTIC protocol for SARS-CoV-2 sequencing (1200 bp amplicons with the midnight primer scheme, see methods for specific modifications)[26]. Live GPU basecalling was performed using Guppy v.5.1.15 (Oxford Nanopore technologies) in high-accuracy mode and the downstream analysis was performed using a modified version of the ARTIC bioinformatics pipeline (https://artic.network/ncov-2019/ncov2019-bioinformatics-sop.html). Briefly, input reads were filtered based on read length and then mapped to the Wuhan-Hu-1 reference genome (accession MN908947.3 [www.ncbi.nlm.nih.gov/bioproject/?term=MN908947.3]) using the 'artic minion' command. BAM alignment files from the ARTIC pipeline were then used for variant calling in longshot (v.0.4.4) with an input VCF file containing VOC Delta and Omicron-BA.1 mutations provided to call variants at specific nucleotide sites. The downstream analysis of VCF files was performed in R v.4.1.3 and involved filtering the VCF file for each sample to exclude mutations shared between both viruses in the mixture (shared, blue), non-lineage-defining mutations (non-LDM, grey), and any sites with overlapping mutations in both viruses that were difficult to call (other, green). Mutations called with a depth of coverage lower than 100 were also excluded from the downstream analysis (bottom left panel). Mutations in filtered VCF files (bottom middle panel) were then used to calculate the mean mutation frequency for each virus per sample. Finally, the mean ± sd virus ratio was calculated for each time point (bottom right panel). **b)** Stacked bar plot showing the frequency of individual mutations called in the VCF file for the WT-614G and Omicron inoculum before (top) and after (bottom) filtering. **c)** Stacked bar plot showing the frequency of individual mutations in the filtered VCF files for all WT-614G and Omicron NEC samples (3 donors, 2 and 6 dpi). These values were used to calculate a mean mutation frequency for each virus per sample, which is shown in **d)**.

Supplementary Figure 2

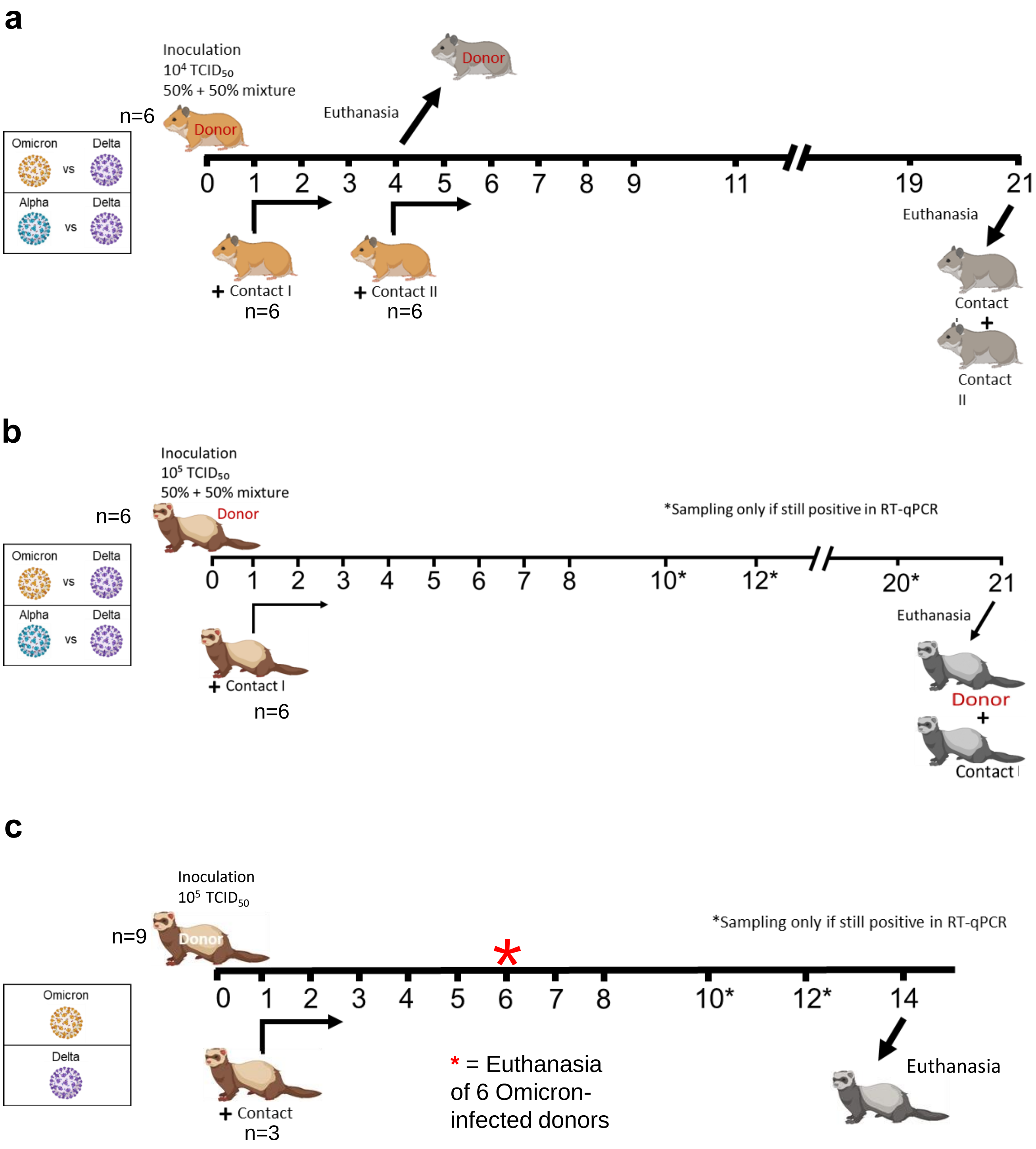

Supplementary Fig. 2: Experimental outline for studies with Syrian hamsters and ferrets. Donor hamsters (n=6) were intranasally inoculated with either an Omicron-BA.1, -Delta or an Alpha-Delta mixture at iso-titer. A Competition studies in hamsters. Each donor was co-housed with one contact I hamster 1dpi. 4dpi after euthanasia of the donors, one contact II hamster was introduced to each contact I hamster. B Study outline for the ferret competition studies with Omicron-BA.1-Delta and Alpha-Delta mixtures. C Timeline for the ferret study with single-variant-inocula (either Omicron-BA.1 or Delta). 6dpi six donor animals, which were inoculated with Omicron-BA.1, were euthanized for determination of viral organ load in URT and LRT.

Supplementary Figure 3

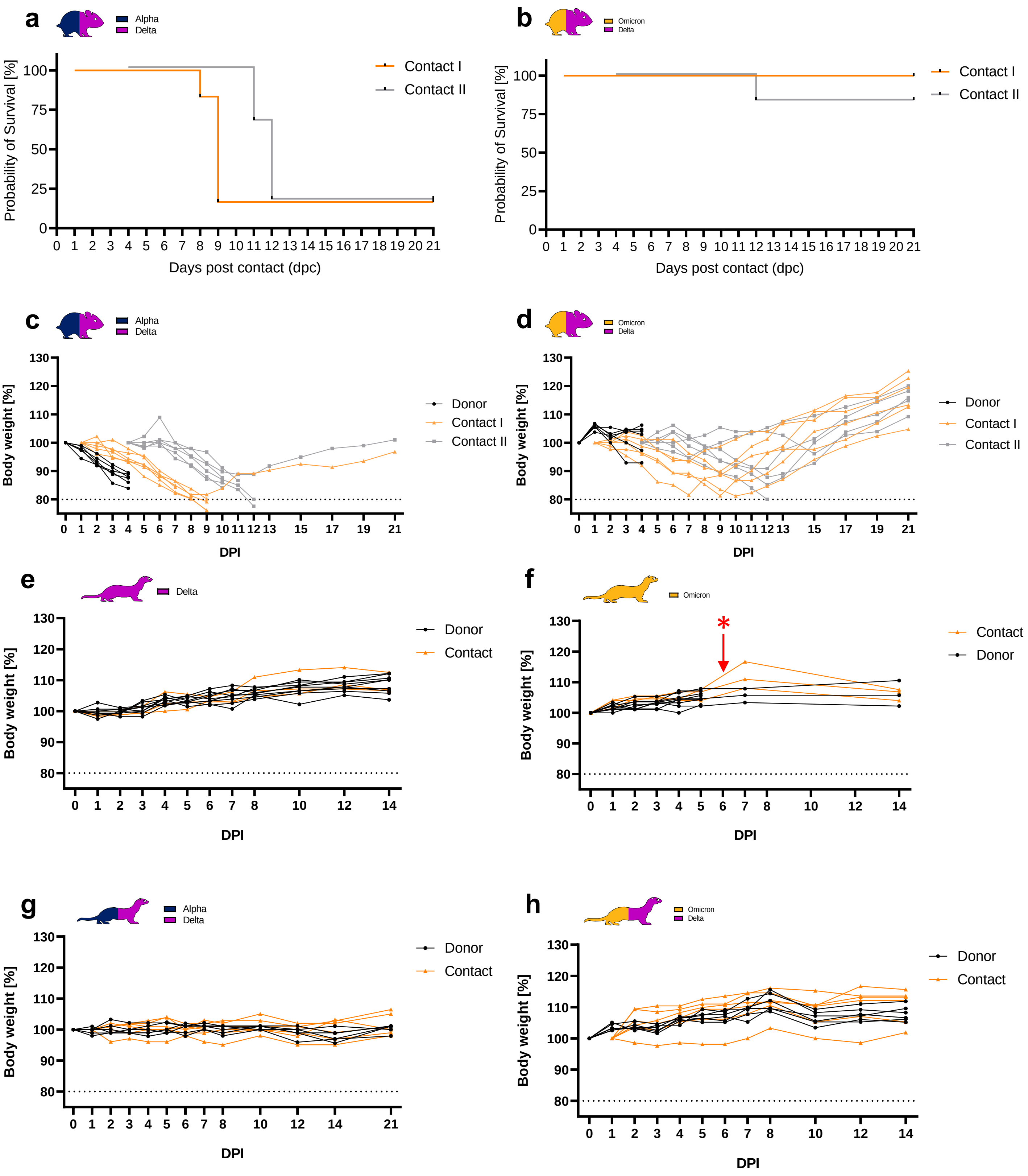

Supplementary Fig. 3: Body weight changes and survival rate of hamsters/ferrets in the competitive infection and transmission experiments. a) Survival of Syrian hamsters during competitive infection and transmission experiment between Alpha and Delta VOC. b) Survival of Syrian hamsters during competitive infection and transmission experiment between Delta and Omicron-BA.1 VOC. c) Percentages of body weight change in Syrian hamsters competitively inoculated with Alpha and Delta VOC. d) Percentages of body weight change in Syrian hamsters competitively inoculated with Delta and Omicron-BA.1 VOC. e) Percentages of body weight change in ferrets during single infection study with Delta VOC. f) Percentages of body weight change in ferrets during single infection study with Omicron-BA.1 VOC. Red star and arrow show timepoint of euthanasia (6 dpi) for six ferrets to analyze viral load distribution in organs. g) Percentages of body weight change in ferrets competitively inoculated with Alpha and Delta VOC. h) Percentages of body weight change in ferrets competitively inoculated with Delta and Omicron-BA.1 VOC.

Supplementary Figure 4

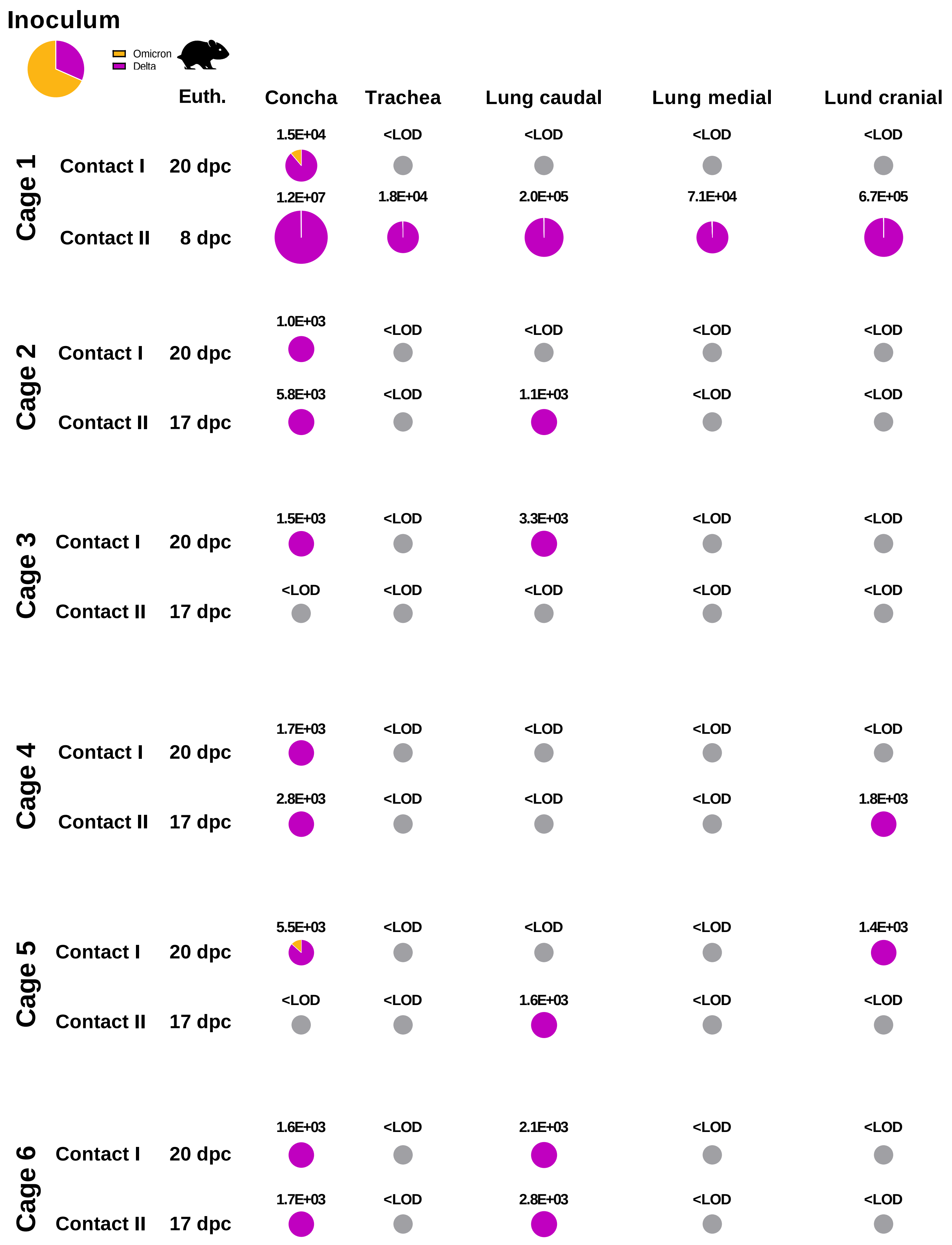

Supplementary Fig. 4: Viral load in organs of Contact I and Contact II hamsters competitively inoculated with Delta and Omicron-BA.1 VOC at respective euthanasia timepoints Viral genome load in upper (URT) and lower (LRT) respiratory tract tissues of Syrian hamsters in the competitive transmission experiment between SARS-CoV-2 VOCs Delta and Omicron-BA.1. Syrian hamsters were inoculated with comparable genome equivalent mixture of Delta and Omicron-BA.1 VOC. Absolute quantification was performed by RT–qPCR analysis of tissue homogenates of Contact I and Contact II hamsters in relation to a set of defined standards. Tissue samples were collected at euthanasia (Euth.). Pie chart colors illustrate the ratio of variants detected in each sample at the indicated dpi or days post contact (dpc). Pie chart sizes are proportional to the total viral genome copies reported above. Grey pies indicate values below the LOD ($<10^3$ viral genome copies per mL).

Supplementary Figure 5

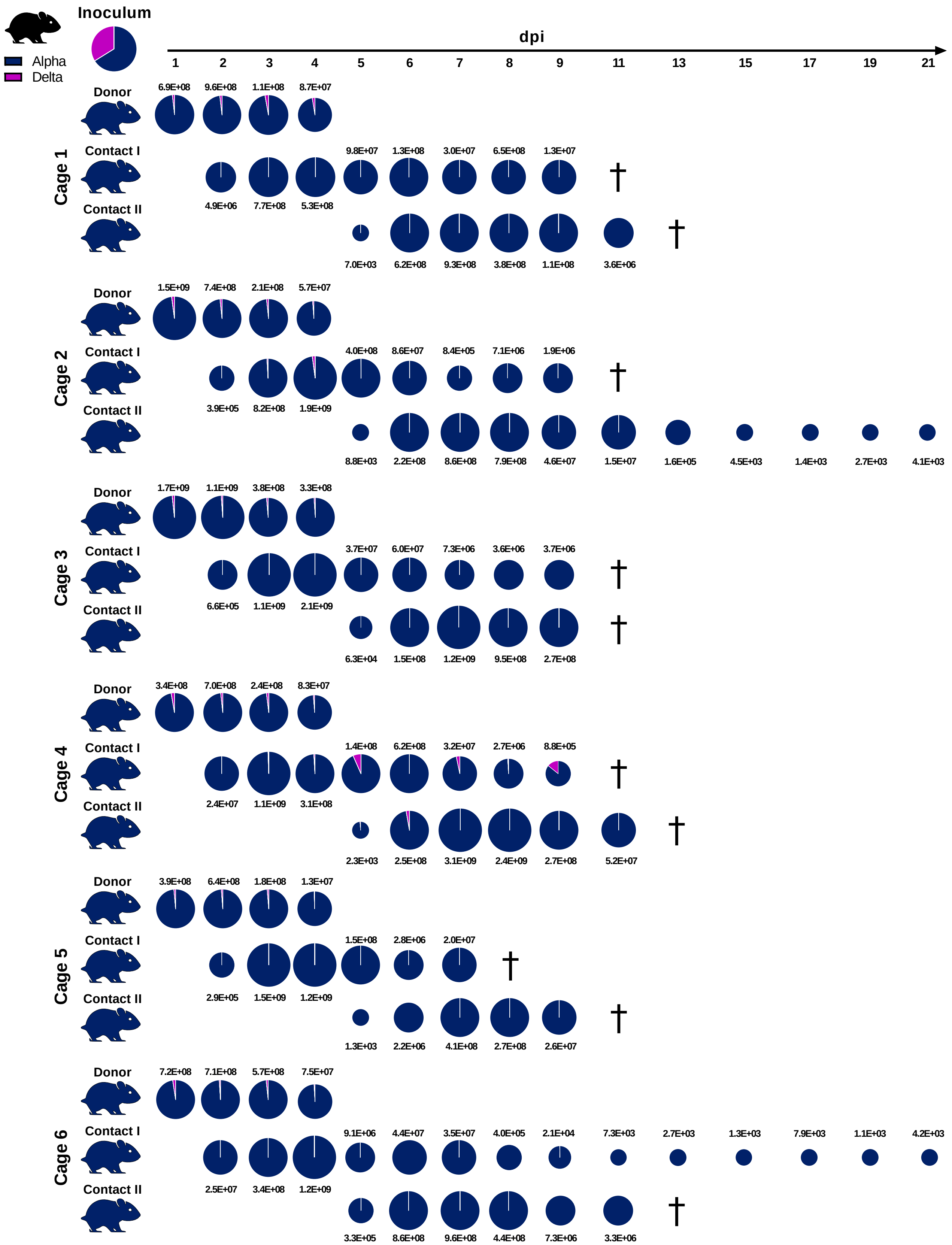

Supplementary Fig. 5: Competitive infection of hamsters with SARS-CoV-2 Delta and Alpha Six donor hamsters were each inoculated intranasally with $10^{4.625}$ $TCID_{50}$ determined by back titration and composed of a mixture of SARS-CoV-2 Alpha (dark blue) and Delta (purple) at 1.95:1 ratio determined by back-titration of the original single virus amounts used in the experiment. Donor hamsters, contact I and II hamsters were co-housed sequentially as shown in Supplementary Data Fig.2. Nasal washings were performed daily from 1-9 dpi and afterwards every two days until 21 dpi. Each pie chart illustrates the ratio of the respective viruses in nasal washings for each sampling day. Total genome copies/mL are indicated above or below the respective pies. Hamster silhouettes are colored according to the dominant variant (>66%) detected in the latest sample of each animal. Black crosses indicate the respective animal was already dead.

Supplementary Figure 6

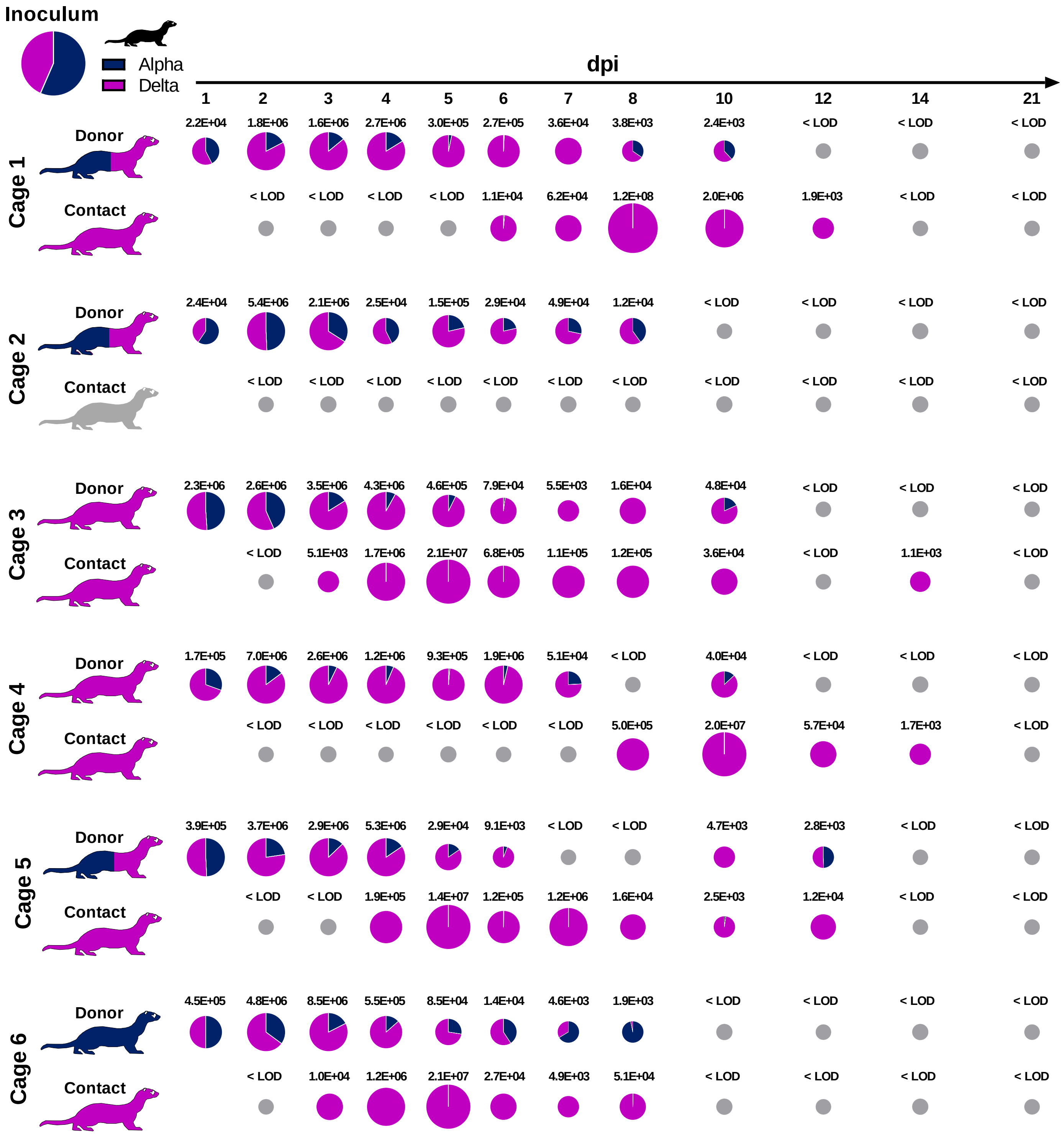

Supplementary Fig. 6: Competitive infection of ferrets with SARS-CoV-2 Alpha and Delta
Six donor ferrets were each inoculated with 10^5 $TCID_{50}$ determined by back titration and composed of a mixture of Alpha (dark blue) and SARS-CoV-2 Delta (purple) at a 1.33:1 ratio determined by back-titration of the original single virus amounts used in the experiment. Donor and Contact ferrets were co-housed sequentially as shown in Supplementary Data Fig.2. Pie charts illustrate the ratio of either SARS-CoV-2 Alpha or SARS-CoV-2 Delta detected in nasal washings of the donor or contact ferrets in the respective ferret groups at indicated dpi. Viral genome copies/mL are shown above or below respective pie charts; Grey pies indicate values below the LOD ($<10^3$ viral genome copies per mL). Coloring of the ferret silhouettes refers to the predominant SARS-CoV-2 variant (>66%) detected in the latest sample of the respective animal.

Supplementary Figure 7

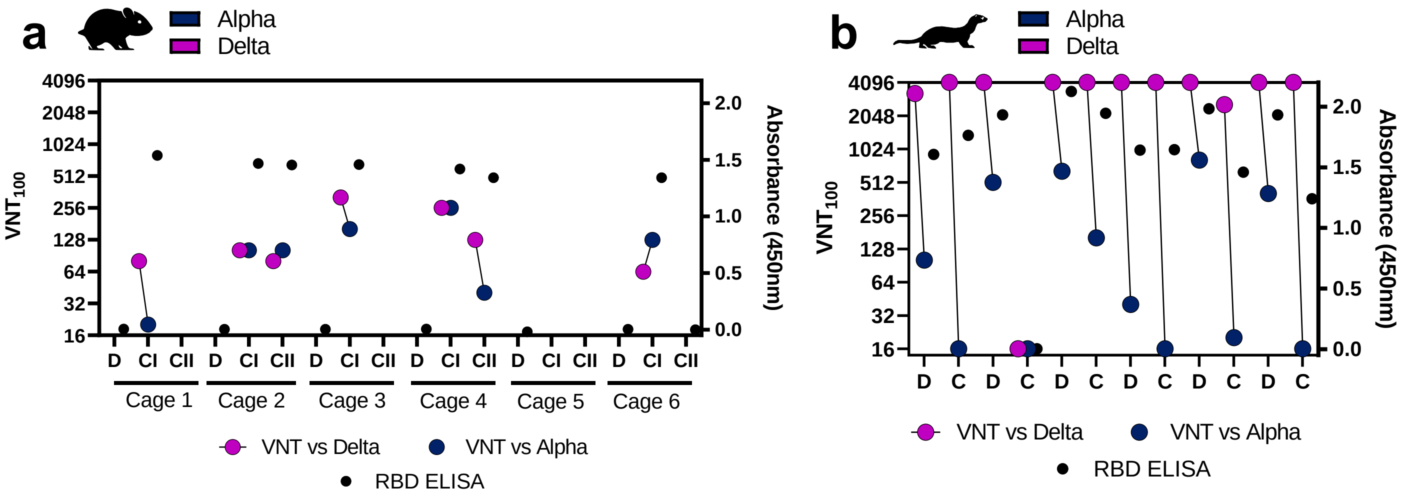

Supplementary Fig. 7: ELISA and VNT100 of sera received from competitive infection experiments with Alpha and Delta in hamsters/ferrets. Blue dots represent neutralization of Alpha variant, purple dots represent neutralization of the Delta variant in the respective animal according to the highest dilution where virus neutralization was visible (left Y-Axis). Black dots show RBD-ELISA-reactivity of animal sera at respective euthanasia timepoint (right Y-Axis) (a) VNT100 and RBD-ELISA from animal sera of the Alpha vs Delta competitive infection and transmission experiment in hamsters (b) VNT100 and RBD-ELISA from animal sera of the competitive infection and transmission experiment with Delta and Alpha VOC in ferrets.

Supplementary Figure 8

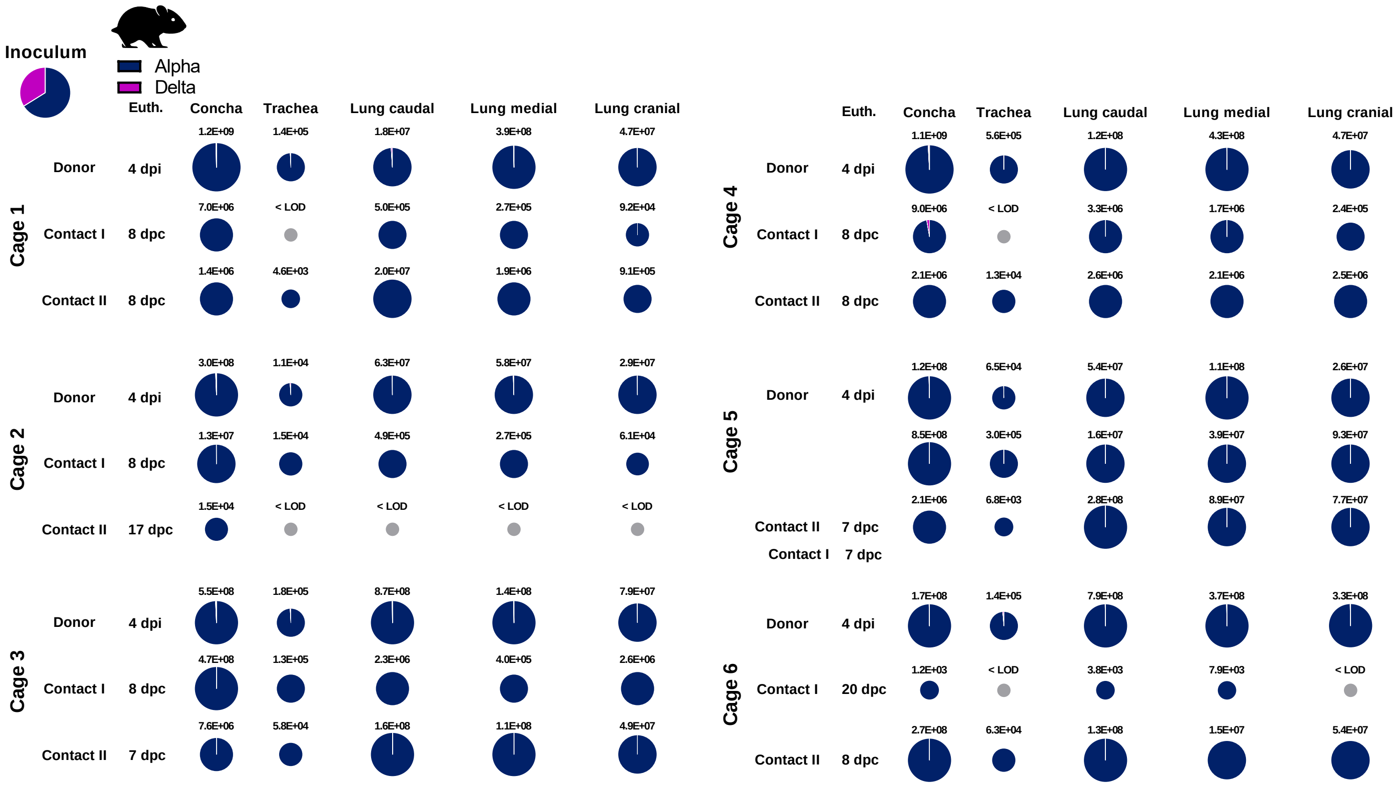

Supplementary Fig. 8: Viral load in organs of Donor, Contact I and Contact II hamsters competitively inoculated with Alpha and Delta VOC at respective euthanasia timepoints. Viral genome load in upper (URT) and lower (LRT) respiratory tract tissues of Syrian hamsters in the competitive transmission experiment between SARS-CoV-2 VOCs Alpha and Delta. Syrian hamsters were inoculated with comparable genome equivalent mixture of either Alpha or Delta VOC. Absolute quantification was performed by RT–qPCR analysis of tissue homogenates of donor, contact I and contact II hamsters in relation to a set of defined standards. Tissue samples were collected at euthanasia (Euth.). Pie chart colors illustrate the ratio of variants detected in each sample at the indicated dpi or days post contact (dpc). Pie chart sizes are proportional to the total viral genome copies reported above. Grey pies indicate values below the LOD (<10^3 viral genome copies per mL).

Supplementary Figure 9

a Single infections with Delta and Omicron

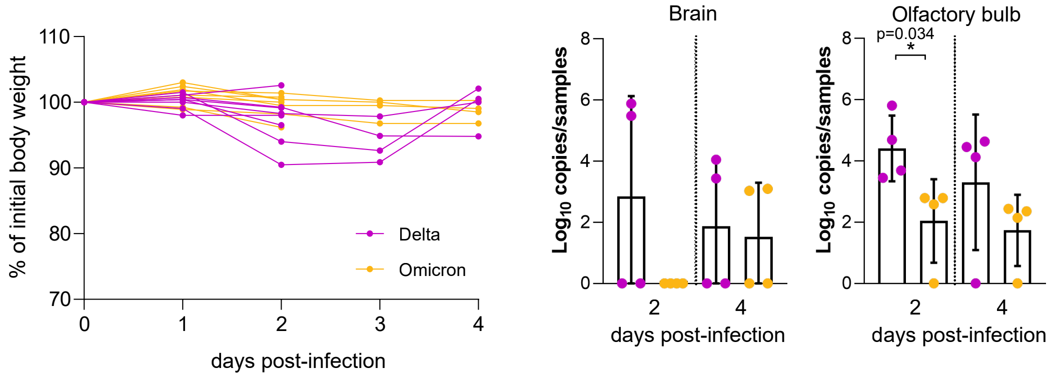

b Competition experiments with Delta, Omicron, S-Delta and S-Omicron

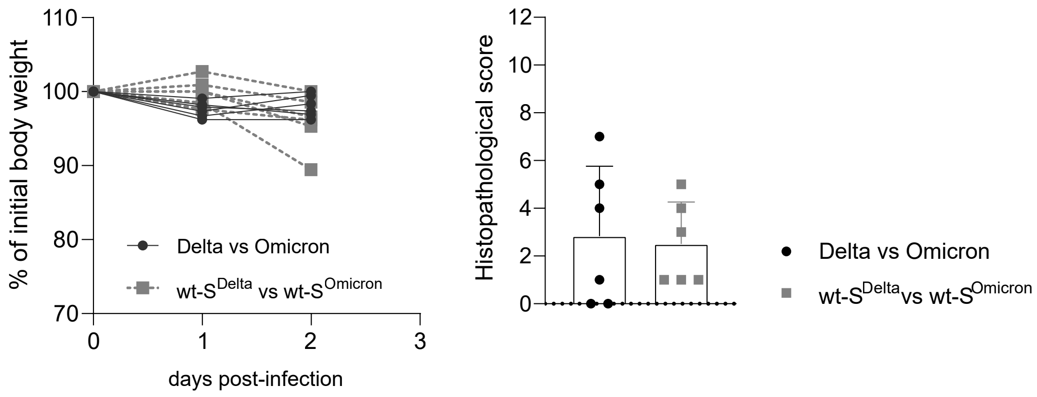

Supplementary Fig. 9: Delta spike mutations drive enhanced fitness in humanized mice. a) hACE2-KI mice (7 to 16 week-old male) were intranasally inoculated with $10^{4.3}$ tissue culture infectious dose 50 ($TCID_{50}$) of Delta or Omicron isolates. The left graph reports the body weight loss for each of the hACE2-KI in Fig. 3a. The right graph depicts the viral copies in brain and olfactory bulb samples quantified using E-gene probe-specific RT–qPCR. Data are mean ± s.d. from the indicated number of biological replicates from a single experiment. Statistical significance was determined using an unpaired two-tailed Student t-test; **P < 0.01. **b)** hACE2-KI mice (7 to 19 week-old female, n=6/group) were intranasally inoculated with 10^4 TCID50 of a 1:1 mix of Delta and Omicron or SARS-CoV-2$^{S\text{-}Delta}$ and SARS-CoV-2$^{S\text{-}Omicron}$. The graph on the left shows the body weight loss for each of the inoculated animal. The graph on the right shows the histopathological score in these mice. Data are mean ± s.d. from the indicated number of biological replicates from a single experiment.

Supplementary Figure 10

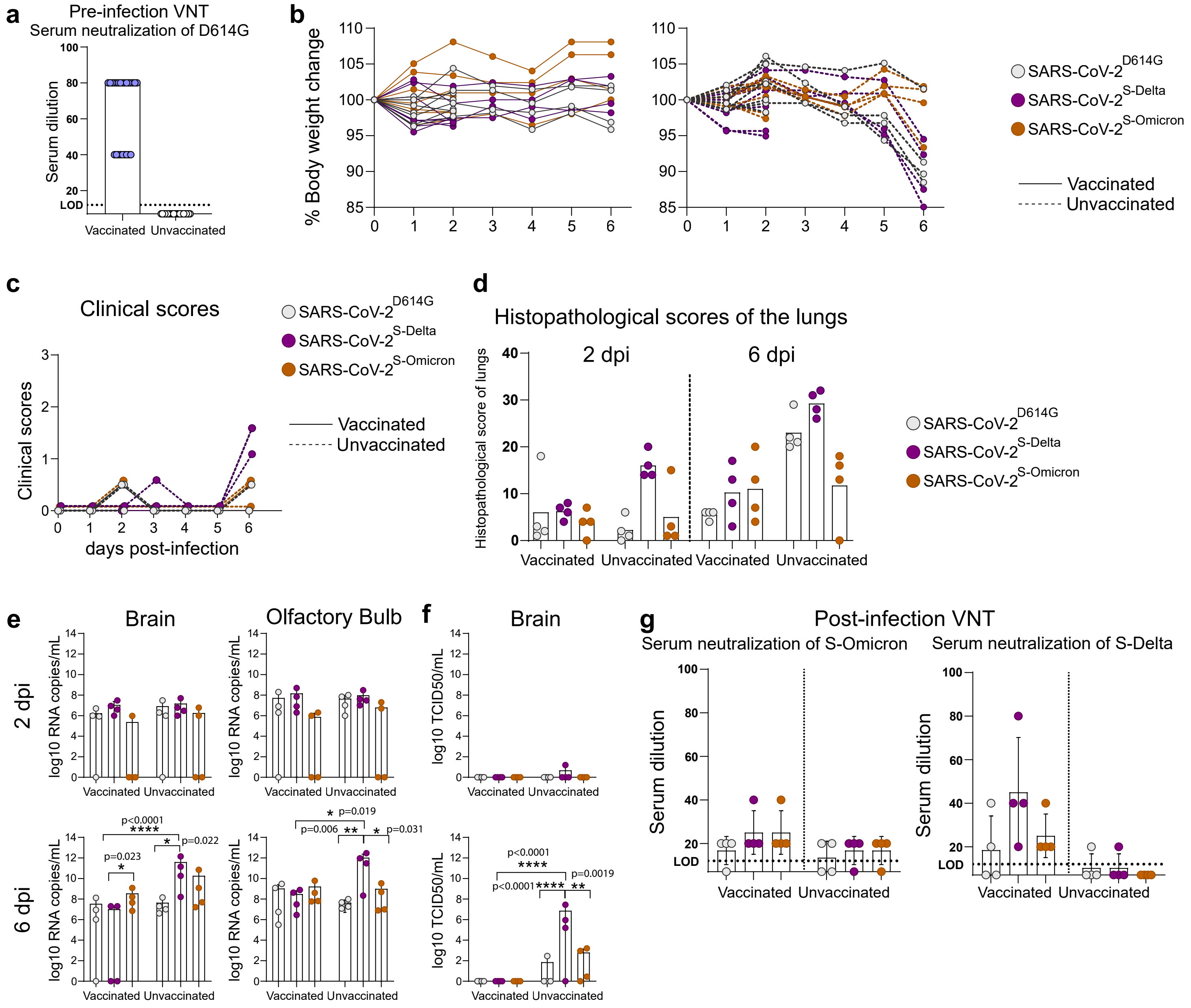

Supplementary Fig. 10: mRNA vaccine induced reduction in replication and pathogenesis of SARS-CoV-2 clones in K18-hACE2 transgenic mice. a) Female K18-hACE2 transgenic mice (7 to 15 weeks old, n=16 mice) were immunized intramuscularly with a single dose of 1 µg of mRNA-Vaccine Spikevax (Moderna). After two weeks the neutralizing antibody titers against SARS-CoV-2^{D614G} were determined. Later, mice were (n=8 mice/group) intranasally inoculated with 10^4 tissue culture infectious dose 50 ($TCID_{50}$) of SARS-CoV-2^{D614G}, SARS-CoV-$2^{S\text{-}Delta}$ and SARS-CoV-$2^{S\text{-}Omicron}$. **b)** The body weight change and **c)** the clinical scores of the mice were monitored daily. **d)** Histopathological scores were given to evaluate the severity of the lung pathology. **e)** Brain and olfactory bulb samples of the infected mice were collected at 2 or 6 days post-infection (dpi) to determine the viral load (n = 4 for each group). Viral RNA-dependent RNA polymerase (RdRp) gene copies of brain and olfactory bulb tissues were quantified using probe-specific RT–qPCR. **f)** Infectious virus titers from the brain samples were determined using $TCID_{50}$ assays in VeroE6/TMPRSS2 cells. **g)** Virus neutralization capacities of the serum collected from infected mice at 6 dpi are tested against SARS-CoV-$2^{S\text{-}Delta}$ and SARS-CoV-$2^{S\text{-}Omicron}$ clones. Each dot in the graphs represents one animal, and the bars show mean values +/- SD. The color key in b also applies to c, d, e and f. Statistical significance was determined using ordinary two-way ANOVA (a–d) and P values were adjusted using Tukey's multiple-comparison test; *$P < 0.05$, **$P < 0.01$, ***$P < 0.001$, ****$P < 0.0001$. Data were obtained from one experiment. Each data point represents one biological replicate.

Supplementary Information Guide

1. **Supplementary Information Table 1:** List of the viruses used in the experiments.
2. **Supplementary Information Table 2:** Histopathological characterization of hACE-2-KI mice infected with Delta or Omicron-BA.1 isolates
3. **Supplementary Information Table 3:** Histopathological characterization of K18-hACE2 mice infected with SARS-CoV-$2^{S\text{-}Delta}$ or SARS-CoV-$2^{S\text{-}Omicron}$
4. **Supplementary Information Table 4:** Primer Pairs for Delta and Omicron.
5. **Supplementary Information Table 5:** Primers and probes for viral RNA detection via RT-qPCR.

Supplementary Information Table 1

SARS-CoV-2	Reference	Model	Inocula
SARS-CoV-2^{D614G}	https://doi.org/10.1038/s41586-021-03361-1	hACE2-K18 mice	SARS-CoV-2^{D614G} Single
		hNEC/hBEC	SARS-CoV-2^{D614G} Single
			SARS-CoV-2^{D614G} vs Delta
			SARS-CoV-2^{D614G} vs Omicron-BA.1
			SARS-CoV-2^{D614G} vs SARS-CoV-2$^{S\text{-}Delta}$
			SARS-CoV-2^{D614G} vs SARS-CoV-2$^{S\text{-}Omicron}$
Alpha	EPI_ISL_751799	Hamster and ferret	Alpha vs Delta
Delta	EPI_ISL_1760647	Ferret	Delta Single
		Hamster and ferret	Alpha vs Delta
			Delta vs Omicron-BA.1
		hNEC/hBEC	Delta Single
			SARS-CoV-2^{D614G} vs Delta
			Delta vs Omicron-BA.1
			Delta vs SARS-CoV-2$^{S\text{-}Delta}$
	EPI_ISL_2535433	hACE2-KI mice	Delta Single
			Delta vs Omicron-BA.1
Omicron-BA.1	EPI_ISL_6959868	Ferret	Omicron-BA.1 Single
		Hamster and ferret	Delta vs Omicron-BA.1
	EPI_ISL_7062525	hACE2-KI mice	Omicron-BA.1 Single
			Delta vs Omicron-BA.1
		hNEC/hBEC	Omicron-BA.1 Single
			SARS-CoV-2^{D614G} vs Omicron-BA.1
			Delta vs Omicron-BA.1
			Omicron-BA.1 vs SARS-CoV-2$^{S\text{-}Omicron}$
SARS-CoV-2$^{S\text{-}Delta}$	Backbone: NCBI Reference Sequence: Wuhan-Hu-1 NC_045512.2<br>Spike: EPI_ISL_5769545	hACE2-KI mice	SARS-CoV-2$^{S\text{-}Delta}$ vs SARS-CoV-2$^{S\text{-}Omicron}$
		NEC/BEC	SARS-CoV-2$^{S\text{-}Delta}$ Single
			SARS-CoV-2^{D614G} vs SARS-CoV-2$^{S\text{-}Delta}$
			Delta vs SARS-CoV-2$^{S\text{-}Delta}$
			SARS-CoV-2$^{S\text{-}Delta}$ vs SARS-CoV-2$^{S\text{-}Omicron}$
		PCLS	SARS-CoV-2$^{S\text{-}Delta}$ vs SARS-CoV-2$^{S\text{-}Omicron}$
SARS-CoV-2$^{S\text{-}Omicron}$	Backbone: NCBI Reference Sequence: Wuhan-Hu-1 NC_045512.2<br>Spike: EPI_ISL_7062525	hACE2-KI mice	SARS-CoV-2$^{S\text{-}Delta}$ vs SARS-CoV-2$^{S\text{-}Omicron}$
		hNEC/hBEC	SARS-CoV-2$^{S\text{-}Omicron}$ Single
			SARS-CoV-2^{D614G} vs SARS-CoV-2$^{S\text{-}Omicron}$
			Omicron-BA.1 vs SARS-CoV-2$^{S\text{-}Omicron}$
			SARS-CoV-2$^{S\text{-}Delta}$ vs SARS-CoV-2$^{S\text{-}Omicron}$
		PCLS	SARS-CoV-2$^{S\text{-}Delta}$ vs SARS-CoV-2$^{S\text{-}Omicron}$

Supplementary Information Table 2

Days post-infection	Delta -Infection								Omicron Infection							
	dpi 2				dpi 4				dpi 2				dpi 4			
Mouse	K1 #13	KI #14	KI #15	KI #16	KI #17	KI #18	KI #19	KI #20	KI #21	KI #22	KI #23	KI #24	KI #25	KI #26	KI #27	KI #28
**Overview**																
Alveolar edema	0	0	0	0	0	0	0	0	0	0	0	0	0	0	0	0
Emphysema	0	0	0	0	0	0	0	0	0	0	0	0	0	0	0	0
Atelectasis	0	0	0	0	0	0	0	0	0	0	0	0	0	0	0	0
**Higher magnification**																
**Inflammation score**																
Alveolar infiltrates	0	0	0	0	0	0	0	0	0	0	0	0	0	0	0	0
Grade	0	0	0	0	0	0	0	0	0	0	0	0	0	0	0	0
Predominant inflammatory cell type	0	0	0	0	0	0	0	0	0	0	0	0	0	0	0	0
Interstitial infiltrates	0	0	0	0	0	1	0	0	0	0	0	0	0	0	0	0
Grade	0	0	0	0	0	4	0	0	0	0	0	0	0	0	0	0
Predominant inflammatory cell type	0	0	0	0	0	M + L	0	0	0	0	0	0	0	0	0	0
Peribronchial infiltrates	0	1	1	0	3	1	1	0	0	1	0	0	0	0	0	0
Grade	0	1	1	0	2	2	2	0	0	1	0	0	0	0	0	0
Predominant inflammatory cell type	0	M + L	M + L	0	M + L	M + L	M + L	0	0	M + L + N	0	0	0	0	0	0
Necrotizing bronchitis	0	3	1	0	3	0	1	0	0	0	0	0	0	0	0	0
Grade	1	2	2	0	2	1	2	2	1	1	1	0	1	1	1	1
Predominant inflammatory cell type	M	M	M + L	0	M	M	M	M	M	M	M	0	M	M	M	M
Intrabronchial mucus increase	0	0	0	0	0	0	0	0	0	0	0	0	0	0	0	0
Perivascular infiltrates	1	0	0	1	0	0	0	1	0	1	0	0	0		0	0
Grade	1	0	0	1	0	0	0	1	0	1	0	0	0	1	0	0
Predominant inflammatory cell type	M + L	0	0	M + L	0	0	0	M + L	0	M + L	0	0	0	L	0	0
**Vascular lesions**																
Intravascular rolling inflammatory cells	0	0	0	0	1	0	0	0	0	0	0	0	0	0	0	0
Endotheliitis	0	0	0	0	1	0	0	0	0	0	0	0	0	0	0	0
Vasculitis	0	0	0	0	0	0	0	0	0	0	0	0	0	0	0	0
Hyaline thrombi	0	0	0	0	0	0	0	0	0	0	0	0	0	0	0	0
Hemosiderin	0	0	0	0	0	0	0	0	0	0	0	0	0	0	0	0
Arterial media hypertrophy/ hyperplasia	0	0	0	0	0	0	0	0	0	0	0	0	0	0	0	0
Vascular occlusion	0	0	0	0	0	0	0	0	0	0	0	0	0	0	0	0
**Diffuse alveolar damage (DAD)/Necrosis**																
Necrosis alveolar epithelial cells (AEC)	0	0	0	0	0	0	0	0	0	0	0	0	0	0	0	0
DAD	0	0	0	0	0	0	0	0	0	0	0	0	0	0	0	0
Intraalveolar fibrin	0	0	0	0	0	0	0	0	0	0	0	0	0	0	0	0
**Varia**																
Hypertrophy/hyperplasia bronchiolar epithelium	0	0	0	0	0	0	0	0	0	0	0	0	0	0	0	0
Hyperplasia /hypertrophy type II pneumocytes	0	0	0	0	0	0	0	0	0	0	0	0	0	0	0	0
Atypical cells/syncytia	0	0	0	0	0	0	0	0	0	0	0	0	0	0	0	0
Pleura	0	0	0	0	0	0	0	0	0	0	0	0	0	0	0	0
BALT	0	0	0	0	0	0	0	0	0	0	0	0	0	0	0	0
TOTAL SCORE	**3**	**7**	**5**	**2**	**12**	**9**	**6**	**4**	**1**	**5**	**1**	**0**	**1**	**3**	**1**	**1**

Days post-infection	Delta / Omicron Mix						SARS-CoV-2$^{S\text{-}Delta}$ / SARS-CoV-2$^{S\text{-}Omicron}$ Mix					
	dpi 2						dpi 2					
Mouse	KI #1	KI #2	KI #3	KI #4	KI #5	KI #6	KI #7	KI #8	KI #9	KI #10	KI #11	KI #12
**Overview**												
Alveolar edema	0	0	0	0	0	0	0	0	0	0	0	0
Emphysema	0	0	0	0	0	0	0	0	0	0	0	0
Atelectasis	0	0	0	0	0	0	0	0	0	0	0	0
**Higher magnification**												
**Inflammation score**												
Alveolar infiltrates	0	0	0	0	0	0	0	0	0	0	0	0
Grade	0	0	0	0	0	0	0	0	0	0	0	0
Predominant inflammatory cell type	0	0	0	0	0	0	0	0	0	0	0	0
Interstitial infiltrates	0	0	0	1	0	0	0	0	0	0	0	0
Grade	0	0	0	2	0	0	0	0	0	0	0	0
Predominant inflammatory cell type	0	0	0	M	0	0	0	0	0	0	0	0
Peribronchial infiltrates	0	1	1	0	0	0	0	0	0	0	0	1
Grade	0	1	1	0	0	0	0	0	0	0	0	1
Predominant inflammatory cell type	0	M + L	M + L + N	0	0	0	0	0	0	0	0	M + L
Necrotizing bronchitis	0	1	3	1	0	0	0	0	0	0	0	0
Grade	0	1	2	1	0	1	1	1	1	1	1	1
Predominant inflammatory cell type	0	M	M	M	0	M	M	M	M	M	M	M
Intrabronchial mucus increase	0	0	0	0	0	0	0	0	0	0	0	0
Perivascular infiltrates	0	0	0	0	0	0	0	1	1	0	0	1
Grade	0	0	0	0	0	0	0	1	2	0	0	1
Predominant inflammatory cell type	0	0	0	0	0	0	0	M + L	M + L	0	0	M + L + N
**Vascular lesions**												
Intravascular rolling inflammatory cells	0	0	0	0	0	0	0	0	0	0	0	0
Endotheliitis	0	0	0	0	0	0	0	0	0	0	0	0
Vasculitis	0	0	0	0	0	0	0	0	0	0	0	0
Hyaline thrombi	0	0	0	0	0	0	0	0	0	0	0	0
Hemosiderin	0	0	0	0	0	0	0	0	0	0	0	0
Arterial media hypertrophy/ hyperplasia	0	0	0	0	0	0	0	0	0	0	0	0
Vascular occlusion	0	0	0	0	0	0	0	0	0	0	0	0
**Diffuse alveolar damage (DAD)/Necrosis**												
Necrosis alveolar epithelial cells (AEC)	0	0	0	0	0	0	0	0	0	0	0	0
DAD	0	0	0	0	0	0	0	0	0	0	0	0
Intraalveolar fibrin	0	0	0	0	0	0	0	0	0	0	0	0
**Varia**												
Hypertrophy/hyperplasia bronchiolar epithelium	0	0	0	0	0	0	0	0	0	0	0	0
Hyperplasia /hypertrophy type II pneumocytes	0	0	0	0	0	0	0	0	0	0	0	0
Atypical cells/syncytia	0	0	0	0	0	0	0	0	0	0	0	0
Pleura	0	0	0	0	0	0	0	0	0	0	0	0
BALT	0	0	#	0	0	0	0	0	0	0	0	0
TOTAL SCORE	**0**	**4**	**7**	**5**	**0**	**1**	**1**	**3**	**4**	**1**	**1**	**5**

Supplementary Information Table 3

	D614G															
	Vaccinated								Unvaccinated							
Days post-infection	dpi 2				dpi 6				dpi 2				dpi 6			
Mouse	K18 #1	K18 #2	K18 #3	K18 #4	K18 #5	K18 #6	K18 #7	K18 #8	K18 #13	K18 #14	K18 #15	K18 #16	K18 #9	K18 #10	K18 #11	K18 #12
Overview																
Alveolar edema	0	0	0	0	0	0	0	0	0	0	0	0	2	0	2	0
Emphysema	0	0	0	0	0	0	0	0	0	0	0	0	0	0	0	0
Atelectasis	0	0	0	0	0	0	0	0	0	0	0	0	2	1	2	2
Higher magnification																
Inflammation score																
Alveolar infiltrates	0	0	0	0	0	0	0	0	0	0	0	0	2	1	2	2
Grade	0	0	0	0	0	0	0	0	0	0	0	0	2	2	2	2
Predominant inflammatory cell type	0	0	0	0	0	0	0	0	0	0	0	0	M + N	M + N	M + N	M + N + L
Interstitial infiltrates	0	0	0	0	0	0	0	0	0	0	0	0	2	1	2	2
Grade	0	0	0	0	0	0	0	0	0	0	0	0	3	3	3	4
Predominant inflammatory cell type	0	0	0	0	0	0	0	0	0	0	0	0	M + L	M + L	M + L	M + L + N
Peribronchial infiltrates	0	0	0	3	1	0	1	1	0	0	0	0	1	1	0	3
Grade	0	0	0	3	2	0	1	1	0	0	0	0	2	3	0	2
Predominant inflammatory cell type	0	0	0	M + L + N	M + L	0	M + L	M + L	0	0	0	0	M + L	M + L	0	M + L
Necrotizing bronchitis	0	0	0	0	0	0	0	0	0	0	0	0	0	0	0	0
Grade	0	1	1	1	1	1	1	1	0	1	1	1	1	1	0	1
Predominant inflammatory cell type	0	M	M	M	M	M	M	M	0	M	M	M	M	M	0	M
Intrabronchial mucus increase	0	2	0	0	0	0	0	2	0	0	0	0	0	0	0	2
Perivascular infiltrates	0	0	0	3	1	1	1	0	0	0	3	0	3	1	3	3
Grade	0	0	0	4	1	1	1	0	0	0	2	0	2	3	2	2
Predominant inflammatory cell type	0	0	0	M + L + N	M + L	M + L	M + L	0	0	0	M + L	0	M + L	M + L	M + L	M + L
Vascular lesions																
Intravascular rolling inflammatory cells	1	0	0	3	0	0	1	0	0	0	0	1	1	3	3	1
Endotheliitis	1	0	0	0	0	1	0	0	0	0	0	0	0	1	1	3
Vasculitis	0	0	0	1	0	0	0	0	0	0	0	0	0	0	0	0
Hyaline thrombi	0	0	0	0	0	0	0	0	0	0	0	0	0	0	0	0
Hemosiderin	0	0	0	0	0	0	0	0	0	0	0	0	0	0	0	0
Arterial media hypertrophy/ hyperplasia	0	0	0	0	0	0	0	0	0	0	0	0	0	0	0	0
Vascular occlusion	0	0	0	0	0	0	0	0	0	0	0	0	0	0	0	0
Diffuse alveolar damage (DAD)/Necrosis																
Necrosis alveolar epithelial cells (AEC)	0	0	0	0	0	0	0	0	0	0	0	0	0	0	0	0
DAD	0	0	0	0	0	0	0	0	0	0	0	0	0	0	0	0
Intraalveolar fibrin	0	0	0	0	0	0	0	0	0	0	0	0	0	0	0	0
Varia																
Hypertrophy/hyperplasia bronchiolar epithelium	0	0	0	0	0	0	0	1	0	0	0	0	0	0	0	0
Hyperplasia /hypertrophy type II pneumocytes	0	0	0	0	0	0	0	0	0	0	0	0	2	2	2	2
Atypical cells/syncytia	0	0	0	0	0	0	0	0	0	0	0	0	0	0	0	0
Pleura	0	0	0	0	0	0	0	0	0	0	0	0	0	0	0	0
BALT	0	0	0	0	0	0	0	0	0	0	0	0	0	0	0	0
**TOTAL SCORE**	**2**	**3**	**1**	**18**	**6**	**4**	**6**	**6**	**0**	**1**	**6**	**2**	**21**	**22**	**20**	**29**

	S-Delta															
	Vaccinated								Unvaccinated							
Days post-infection	dpi 2				dpi 6				dpi 2				dpi 6			
Mouse	K18 #17	K18 #18	K18 #19	K18 #20	K18 #21	K18 #22	K18 #23	K18 #24	K18 #25	K18 #26	K18 #27	K18 #28	K18 #29	K18 #30	K18 #31	K18 #32
Overview																
Alveolar edema	0	0	0	0	0	0	0	0	0	0	0	0	2	2	2	0
Emphysema	0	0	0	0	0	0	0	0	0	0	0	0	0	0	0	0
Atelectasis	0	0	0	0	0	0	0	0	1	1	1	1	2	2	0	3
Higher magnification																
Inflammation score																
Alveolar infiltrates	0	0	0	0	0	0	0	0	1	0	0	0	1	2	2	3
Grade	0	0	0	0	0	0	0	0	2	0	0	0	2	2	3	2
Predominant inflammatory cell type	0	0	0	0	0	0	0	0	M + L	0	0	0	M + N + L	M + L + N	M + N + L	M + N
Interstitial infiltrates	0	0	0	0	0	0	0	0	1	1	1	1	2	2	2	3
Grade	0	0	0	0	0	0	0	0	3	3	3	3	4	4	4	4
Predominant inflammatory cell type	0	0	0	0	0	0	0	0	M + L	M + L	M + L + N	M + L	M + L	M + L	M + L	M + L
Peribronchial infiltrates	1	1	1	0	1	3	3	3	0	1	1	1	3	3	3	3
Grade	3	1	1	0	1	3	4	2	0	1	1	1	2	2	2	4
Predominant inflammatory cell type	M + L	M + L	M + L	0	M + L	M + L + N	M + L	M + L + N	0	M + L	M + L	M + L	M + L	M + L	M + L	M + L
Necrotizing bronchitis	0	0	0	0	0	0	0	0	0	0	0	0	0	0	0	0
Grade	0	2	1	1	1	1	0	1	1	1	1	1	1	2	2	1
Predominant inflammatory cell type	0	M	M	M	M	M	0	M	M	M	M	M	M	M	M	M
Intrabronchial mucus increase	0	0	0	0	0	0	0	0	0	0	0	0	0	0	0	0
Perivascular infiltrates	1	1	1	1	0	3	3	1	3	1	1	1	3	3	3	3
Grade	1	1	3	1	0	4	3	1	1	3	2	2	2	3	4	3
Predominant inflammatory cell type	M + L	M + L	M + L	M + L + N	0	M + L + N	M + L	M + L	M + L	M + L	M + L	M + L	M + L	M + L	M + L	M + L
Vascular lesions																
Intravascular rolling inflammatory cells	0	1	1	0	0	3	0	0	3	3	3	1	3	0	3	3
Endotheliitis	0	0	0	0	0	0	0	0	1	1	0	1	3	1	1	0
Vasculitis	0	0	0	0	0	0	0	0	3	0	0	1	0	0	0	0
Hyaline thrombi	0	0	0	0	0	0	0	0	0	0	0	0	0	0	0	0
Hemosiderin	0	0	0	0	0	0	0	0	0	0	0	0	0	0	0	0
Arterial media hypertrophy/ hyperplasia	0	0	0	0	0	0	0	0	0	0	0	0	0	0	0	0
Vascular occlusion	0	0	0	0	0	0	0	0	0	0	0	0	0	0	0	0
Diffuse alveolar damage (DAD)/Necrosis																
Necrosis alveolar epithelial cells (AEC)	0	0	0	0	0	0	0	0	0	0	0	0	0	0	0	0
DAD	0	0	0	0	0	0	0	0	0	0	0	0	0	0	0	0
Intraalveolar fibrin	0	0	0	0	0	0	0	0	0	0	0	0	0	0	0	0
Varia																
Hypertrophy/hyperplasia bronchiolar epithelium	0	0	0	1	0	0	0	0	0	0	0	0	0	0	0	0
Hyperplasia /hypertrophy type II pneumocytes	0	0	0	0	0	0	0	0	1	1	1	1	2	2	2	3
Atypical cells/syncytia	0	0	0	0	0	0	0	0	0	0	0	0	0	0	0	0
Pleura	0	0	0	0	0	0	0	0	0	0	0	0	0	0	0	0
BALT	0	0	0	0	0	0	0	0	0	0	0	0	0	0	0	0
**TOTAL SCORE**	**6**	**7**	**8**	**4**	**3**	**17**	**13**	**8**	**20**	**16**	**14**	**14**	**28**	**26**	**31**	**32**

	S-Omicron															
	Vaccinated								Unvaccinated							
Days post-infection	dpi 2				dpi 6				dpi 2				dpi 6			
Mouse	K18 #37	K18 #38	K18 #39	K18 #40	K18 #33	K18 #34	K18 #35	K18 #36	K18 #41	K18 #42	K18 #43	K18 #44	K18 #45	K18 #46	K18 #47	K18 #48
Overview																
Alveolar edema	0	0	0	0	0	1	0	0	0	0	0	0	0	1	0	0
Emphysema	0	0	0	0	0	0	0	0	0	0	0	0	0	0	0	0
Atelectasis	0	0	0	0	1	0	0	1	0	0	0	1	1	1	0	1
Higher magnification																
Inflammation score																
Alveolar infiltrates	0	0	0	0	1	0	0	0	0	0	0	1	0	0	0	0
Grade	0	0	0	0	1	0	0	0	0	0	0	4	0	0	0	0
Predominant inflammatory cell type	0	0	0	0	M	0	0	0	0	0	0	M + N	0	0	0	0
Interstitial infiltrates	0	0	0	0	2	0	0	1	0	0	0	1	1	1	0	1
Grade	0	0	0	0	3	0	0	3	0	0	0	3	3	1	0	3
Predominant inflammatory cell type	1	0	0	0	M + L	0	0	M + L	0	0	0	M + L	M + L	M + L	0	M + L
Peribronchial infiltrates	2	1	0	0	3	3	1	1	0	0	0	1	1	1	0	1
Grade	M + L + N	1	0	0	3	2	1	1	0	0	0	3	2	2	0	3
Predominant inflammatory cell type	0	M + L	0	0	M + L + N	M + L	M + L	M + L	0	0	0	M + L	M + L	M + L	0	M + L
Necrotizing bronchitis	0	0	0	0	0	0	0	0	0	0	0	0	0	0	0	0
Grade	0	0	0	1	0	0	1	0	1	1	1	1	2	2	0	1
Predominant inflammatory cell type	0	0	0	M	0	0	M	0	M	M	M	M	M	M	0	M
Intrabronchial mucus increase	0	0	0	0	0	0	0	0	0	0	0	0	0	0	0	0
Perivascular infiltrates	0	1	0	1	1	1	0	3	0	0	1	0	3	3	0	1
Grade	0	3	0	2	3	1	0	2	0	0	1	0	3	1	0	2
Predominant inflammatory cell type	0	M + L + P	0	M + L	M + L + N	M + L	0	M + L	0	0	M + L	0	M + L	M + L + P	0	M + L
Vascular lesions																
Intravascular rolling inflammatory cells	1	1	0	0	1	0	1	1	0	0	0	0	1	1	0	3
Endotheliitis	0	0	0	0	1	0	0	0	0	0	0	0	0	0	0	0
Vasculitis	0	0	0	0	0	0	0	0	0	0	0	0	0	0	0	0
Hyaline thrombi	0	0	0	0	0	0	0	0	0	0	0	0	0	0	0	0
Hemosiderin	0	0	0	0	0	0	0	0	0	0	0	0	0	0	0	0
Arterial media hypertrophy/ hyperplasia	0	0	0	0	0	0	0	0	0	0	0	0	0	0	0	0
Vascular occlusion	0	0	0	0	0	0	0	0	0	0	0	0	0	0	0	0
Diffuse alveolar damage (DAD)/Necrosis																
Necrosis alveolar epithelial cells (AEC)	0	0	0	0	0	0	0	0	0	0	0	0	0	0	0	0
DAD	0	0	0	0	0	0	0	0	0	0	0	0	0	0	0	0
Intraalveolar fibrin	0	0	0	0	0	0	0	0	0	0	0	0	0	0	0	0
Varia																
Hypertrophy/hyperplasia bronchiolar epithelium	0	0	0	0	0	0	0	0	0	0	0	0	1	0	0	0
Hyperplasia /hypertrophy type II pneumocytes	0	0	0	0	1	0	0	1	0	0	0	1	1	1	0	1
Atypical cells/syncytia	0	0	0	0	0	0	0	0	0	0	0	0	0	0	0	0
Pleura	0	0	0	0	0	0	0	0	0	0	0	0	0	0	0	0
BALT	0	0	0	0	0	0	0	0	0	0	0	0	0	0	0	0
**TOTAL SCORE**	**4**	**7**	**0**	**4**	**20**	**7**	**4**	**13**	**1**	**1**	**3**	**15**	**18**	**13**	**0**	**16**

Supplementary Information Table 4

Spike-Variant	Primer name	Primer sequence (5' to 3')	Amplification product (bp)	PCR Template (cDNA or YAC from previous clones)
SARS-CoV-2S-Delta	WU-19-F	GGAGTCACATTAATTGGAGAAGC	1537	SpD614G
	CoV2-Sp-T19R-R	GTATGCAGGGGGTAATTGAGTTCTGGTTcTAAGATTAACACACTGACTAG		
	CoV2-Sp-T19R-F	CTAGTCAGTGTGTTAATCTTAgAACCAGAACTCAATTACCCCCTGCATAC	269	SpD614G
	CoV2-Sp-T95I-R	TATTATGTTAGACTTCTCAaTGGAAGCAAAATAAACACCATCATTAAATG		
	CoV2-Sp-T95I-F	TTTAATGATGGTGTTTATTTTGCTTCCAtTGAGAAGTCTAACATAATAAG	234	SpG142D
	CoV2-Sp-E156d-E157d-R158G-R1+R2	GCAATTATTCGCACTAGAATAAACTCcACTTTCCATCCAACTTTTGTTG		
	CoV2-Sp-E156d-E157d-R158G-F1+F2	CAAAAACAACAAAAGTTGGATGGAAAGTgGAGTTTATTCTAGTGCG	1199	SpL452R
	CoV2-Sp-T478K-R	CCTTCAACACCATTACAAGGTtTGCTACCGGCCTGATAGATTTCAGTTG		
	CoV2-Sp-T478K-F	CAACTGAAATCTATCAGGCCGGTAGCAaACCTTGTAATGGTGTTGAAGG	1468	SpD614G
	CoV2-Sp-D950N-R	GCTTGTGCATTTTGGTTGACCACATtTTGAAGTTTTCCAAGTGCACTTGC		
	CoV2-Sp-D950N-F	GCAAGTGCACTTGGAAAACTTCAAaATGTGGTCAACCAAAATGCACAAGC	1555	SpD614G
	WU-22-R	TCATGTTCAGAAATAGGACTTGTTG		
SARS-CoV-2$^{S\text{-}Omicron}$	WU-19-F	GGAGTCACATTAATTGGAGAAGC	1203	SpD614G
	WU-60-R	TTGTTCGCGTGGTTTGCCAAG		
	WU-61-F	CTTGGAATGCTGATCTTTATAAGC	1221	cDNA of Omicron for Omicron spike and OmNTD; SpD614G for OmRBD and OmCS
	WU-62-R	TAGAAAAGTCCTAGGTTGAAGATAAC		
	WU-63-F	TTCGGCTTTAGAACCATTGGTAG	1209	cDNA of Omicron for Omicron spike and OmRBD; SpD614G for OmNTD and OmCS
	CoV2-D614G-R	GCAACAGGGACTTCTGTGCAGTTAACACCCTGATAAAGAACAGCAACCTG		
	CoV2-D614G-F	CAGGTTGCTGTTCTTTATCAGGGTGTTAACTGCACAGAAGTCCCTGTTGC	815	cDNA of Omicron for Omicron spike and OmCS ; SpD614G for OmNTD and OmRBD
	WU-64-R	AACAGTGCAGAAGTGTATTGAGC		
	WU-65-F	TGATTGCCTTGGTGATATTGCTG	1206	cDNA of Omicron for Omicron spike; SpD614G for OmNTD, OmRBD, OmCS
	WU-66-R	CAACTGGTCATACAGCAAAGCAT		
	WU-67-F	GACATCTCTGGCATTAATGCTTC	877	SpD614G
	WU-22-R	TCATGTTCAGAAATAGGACTTGTTG		

Supplementary Information Table 5

Assay Name	Oligo name	Sequence (5´- 3´)	Conc.	Position
SARS-CoV-2-Alpha-S assay	Alpha-S-22011-F	AAA GTT GGA TGG AAA GTG AGT TCA	10 µM	22011
	SARS2-S-22132R	CCT AAG ATT TTT GAA ATT ACC CTG T	10 µM	22132
	SARS2-S-22074FAM	FAM- TCT CTC AGC CTT TTC TTA TGG ACC T -BHQ1	5 µM	22074
SARS-CoV-2-Delta-S assay	Delta-S-22011-F	AAA GTT GGA TGG AAA GTG GAG	10 µM	22011
	SARS2-S-22132R	CCT AAG ATT TTT GAA ATT ACC CTG T	10 µM	22132
	SARS2-S-22074FAM	FAM- TCT CTC AGC CTT TTC TTA TGG ACC T -BHQ1	5 µM	22074
SARS-CoV-2-Omicron-BA.1-S assay	O-S22172-F	TATTCTAAGCACACGCCTATTATAG	10 µM	22172
	O-S22280-R	TAGTGATGTTAATACCTATTGGCAAATC	10 µM	22280
	O-S22202-FAM	FAM-CGTGAGCCAGAAGATCTCCCTC-BHQ1	5 µM	22202
