# Supplementary material for: The spike gene is a major determinant for the SARS-CoV-2 Omicron-BA.1 phenotype
Source: Nat Commun. 2022 Oct 7;13:5929. doi: 10.1038/s41467-022-33632-y (PMC9543931; doi:10.1038/s41467-022-33632-y)
Supplement: Supplementary file 3 — Source Data [file 41467_2022_33632_MOESM3_ESM.zip › 3. Source data files/SourceData_Guide_Final_220824.docx]

**Guide for Source Data file**

**Title: The spike gene is a major determinant for the SARS-CoV-2 Omicron-BA.1 phenotype**

Short title: SARS-CoV-2 spike drives Omicron phenotype

G.Tuba Barut^1,2^†, Nico Joel Halwe^3^†, Adriano Taddeo^1,2^†, Jenna N. Kelly^1,2,4,5^†, Jacob Schön^3^, Nadine Ebert^1,2^, Lorenz Ulrich^3^, Christelle Devisme^1,2^, Silvio Steiner^1,2^, Bettina Salome Trüeb^1,2^, Bernd Hoffmann^3^, Inês Berenguer Veiga^1,2^, Nathan Georges François Leborgne^1,2^, Etori Aguiar Moreira^1,2^, Angele Breithaupt^6^, Claudia Wylezich^3^, Dirk Höper^3^, Kerstin Wernike^3^, Aurélie Godel^1,2^, Lisa Thomann^1,2^, Vera Flück^1,2^, Hanspeter Stalder^1,2^, Melanie Brügger^1,2^, Blandina I. Oliveira Esteves^1,2^, Beatrice Zumkehr^1,2^, Guillaume Beilleau^1,2,7^, Annika Kratzel^1,2^, Kimberly Schmied^1,2^, Sarah Ochsenbein^1,2^, Reto M. Lang^1,2,7^, Manon Wider^8,^, Carlos Machahua^9,10^, Patrick Dorn^11,12^, Thomas M. Marti^11,12^, Manuela Funke-Chambour^9,10^, Andri Rauch^4,13^, Marek Widera^14^, Sandra Ciesek^14^, Ronald Dijkman^4,5,8^, Donata Hoffmann^3^, Marco P. Alves^1,2,4^‡*, Charaf Benarafa^1,2,4^‡*, Martin Beer^3,5^‡*,Volker Thiel^1,2,4,5^‡*

Affiliations:

*^1^Institute of Virology and Immunology, Bern and Mittelhäusern, Switzerland*

*^2^Department of Infectious Diseases and Pathobiology, Vetsuisse Faculty, University of Bern, Bern, Switzerland*

*^3^Institute of Diagnostic Virology, Friedrich-Loeffler-Institut, Greifswald-Insel Riems, Germany*

*^4^Multidisciplinary Center for Infectious Diseases, University of Bern, Bern, Switzerland*

*^5^European Virus Bioinformatics Center, Jena, Germany*

*^6^Department of Experimental Animal Facilities and Biorisk Management, Friedrich-Loeffler-Institut, Greifswald-Insel Riems, Germany*

*^7^Graduate School for Cellular and Biomedical Sciences, University of Bern, Bern, Switzerland*

*^8^Institute for Infectious Diseases, University of Bern, Bern, Switzerland*

*^9^Department of Pulmonary Medicine, Inselspital, Bern University Hospital, University of Bern, Bern, Switzerland*

*^10^Department for Pulmonary Medicine, BioMedical Research, University of Bern, Bern, Switzerland*

*^11^Division of General Thoracic Surgery, Inselspital, Bern University Hospital, University of Bern, Bern, Switzerland*

*^12^Department for BioMedical Research, Inselspital, Bern University Hospital, University of Bern, Switzerland*

*^13^Department of Infectious Diseases, Inselspital, Bern University Hospital, University of Bern, Switzerland*

*^14^Institute of Medical Virology, University Hospital Frankfurt, Goethe University, Frankfurt am Main, Germany*

*†These authors contributed equally to this work.*

*‡These authors jointly lead this work.*

**Corresponding authors.*

*Volker Thiel; e-mail:* [*volker.thiel@vetsuisse.unibe.ch*](mailto:volker.thiel@vetsuisse.unibe.ch)*; phone: +41-31-6312413*

*Charaf Benarafa; e-mail: [charaf.benarafa@vetsuisse.unibe.ch](mailto:charaf.benarafa@vetsuisse.unibe.ch); phone: +41-58-4699246*

*Martin Beer; e-mail:* [*martin.beer@fli.de*](mailto:martin.beer@fli.de)*; phone: +49-38-35171200*

*Marco P. Alves; [e-mail:marco.alves@vetsuisse.unibe.ch](mailto:e-mail:marco.alves@vetsuisse.unibe.ch)*

**List of files provided:**

## Source Data Fig 1: Raw data for Figure 1.

### Sheet 1: Overview of Delta and Omicron-BA.1 mutations. Table of mutations found in VOC Delta and Omicron-BA.1, including nucleotide positions, amino acid changes, and mutation type.

### Sheet 2: Plaques sizes and statistical analysis. Table showing plaque sizes that were measured for each virus and the results from statistical testing.

### Sheet 3: Kinetics hNEC, TCID50 data and statistical analysis. TCID50 results for each virus in hNECs and corresponding statistical analysis for each time point.

### Sheet 4: Kinetics hBEC, TCID50 data and statistical analysis. TCID50 results for each virus in hBECs and corresponding statistical analysis for each time point.

### Sheet 5: Mean virus ratio for competition assays, hNEC and hBEC.

### Sheet 6: Mean virus ratio for competition assays, lung explants.

## Source Data 2 Fig 2: Raw data for Figure 2.

### Sheet 1: Viral genome copy numbers per mL of nasal washings determined by variant specific RT-qPCR of Delta and Omicron-BA.1 competitively infected hamsters.

### Sheet 2: Viral genome copy numbers per mL in respiratory organs determined by variant specific RT-qPCR of Delta and Omicron-BA.1 competitively inoculated hamsters.

### Sheet 3: Viral genome copy numbers per mL of nasal washings determined by variant specific RT-qPCR of Delta and Omicron-BA.1 competitively inoculated ferrets.

### Sheet 4: Serological analysis of Delta and Omicron-BA.1 competitively inoculated hamsters.

### Sheet 5: Serological analysis of Delta and Omicron-BA.1 competitively inoculated ferrets.

### Sheet 6: Viral genome copy numbers per mL of nasal washings determined by variant specific RT-qPCR and serological analysis of Delta and Omicron-BA.1 single infected ferrets.

## Source Data Fig 3: Raw data for Figure 3.

### Sheet 1: Results relative to the single infection of hACE2-KI mice Delta or Omicron-BA.1 isolates (body weight loss; viral RNA copy numbers per mL of oropharyngeal swabs or per lung and nose samples determined by E-gene RT-qPCR; viral titers in lung and nose by TCID_50_ assay and histopathological score).

### Sheet 2: Results relative to competition experiments in hACE2-KI mice competitively inoculated with Delta/Omicron-BA.1 mix (TCID_50_ and viral RNA copy numbers of each VOC in the infection inoculum; Viral RNA copy numbers per mL of oropharyngeal swab or sample nasal washings determined by variant specific RT-qPCR)

### Sheet 3: Results relative to competition experiments in hACE2-KI mice competitively inoculated with SARS-CoV-2^S-Delta^/SARS-CoV-2^S-Omicron^ mix (TCID_50_ and viral RNA copy numbers of each VOC in the infection inoculum; Viral RNA copy numbers per mL of oropharyngeal swab or sample nasal washings determined by variant specific RT-qPCR)

## Source Data Fig 4: Raw data for Figure 4.

### Sheet 1: Tables showing body weight records of K18 mouse experiment.

### Sheet 2: Viral genome copy numbers per ml by RT-qPCR and statistics for K18 mouse experiment.

**VII.** **Source Data Supplementary Fig 3: Raw Data for Supplementary Figure 3**

### Sheet 1: Body weight change of Alpha and Delta competitively infected hamsters

### Sheet 2: Body weight change of Delta and Omicron-BA.1 competitively infected hamsters

### Sheet 3: Body weight change of Delta single infected ferrets

### Sheet 4: Body weight change of Omicron-BA.1 single infected ferrets

### Sheet 5: Body weight change of Alpha and Delta competitively infected ferrets

### Sheet 6: Body weight change of Delta and Omicron-BA.1 competitively infected ferrets

**VIII.** **Source Data Supplementary Fig 4: Raw Data for Supplementary Figure 4**

### Sheet 1: Viral genome copy numbers per mL in respiratory organs determined by variant specific RT-qPCR of Delta and Omicron-BA.1 competitively inoculated hamsters

**IX.** **Source Data Supplementary Fig 5: Raw Data for Supplementary Figure 5**

### Sheet 1: Viral genome copy numbers per mL in respiratory organs determined by variant specific RT-qPCR of Alpha and Delta competitively inoculated hamsters

**X.** **Source Data Supplementary Fig 6: Raw Data for Supplementary Figure 6**

### Sheet 1: Viral genome copy numbers per mL in respiratory organs determined by variant specific RT-qPCR of Alpha and Delta competitively inoculated ferrets

**XI.** **Source Data Supplementary Fig 7: Raw Data for Supplementary Figure 7**

### Sheet 1: Serological analysis of Alpha and Delta competitively inoculated hamsters

### Sheet 2: Serological analysis of Alpha and Delta competitively inoculated ferrets

**XII.** **Source Data Supplementary Fig 8: Raw Data for Supplementary Figure 8**

### Sheet 1: Viral genome copy numbers per mL in respiratory organs determined by variant specific RT-qPCR of Delta and Omicron-BA.1 competitively inoculated hamsters.

**XIII.** **Source Data Supplementary Fig 9: Raw Data for Supplementary Figure 9**

### Sheet 1: Results relative to the single infection of hACE2-KI mice Delta or Omicron-BA.1 isolates (body weight loss for each of the hACE2-KI in Figure 3a; viral RNA copy numbers per brain and olfactory bulb samples determined by E-gene RT-qPCR).

### Sheet 2: Results relative to competition experiments in hACE2-KI mice competitively inoculated with Delta/Omicron-BA.1 mix or SARS-CoV-2^S-Delta^/SARS-CoV-2^S-Omicron^ mix (body weight loss for each of the inoculated hACE2-KI and histopathological score)

**XIV.** **Source Data Supplementary Fig 10: Raw Data for Supplementary Figure10**

### Sheet 1: Tables showing clinical scoring records of K18 mouse experiment.

### Sheet 2: Histopathological score for K18 mouse experiment.

### Sheet 3: Viral genome copy numbers per ml by RT-qPCR for K18 mouse experiment.

### Sheet 4: TCID_50_/ml by RT-qPCR for K18 mouse experiment.
